# Trend, disparities, and projection analysis of public data on shoulder fractures in Sweden: a retrospective analysis of two hundred and sixty two thousand, four hundred and forty four fractures

**DOI:** 10.1007/s00264-024-06287-1

**Published:** 2024-09-11

**Authors:** Martin Magnéli, Michael Axenhus

**Affiliations:** 1 Department of Orthopaedic Surgery, Danderyd Hospital, Stockholm, Sweden; 2 Department of Clinical Sciences at Danderyd Hospital, Karolinska Institutet, Stockholm, Sweden

**Keywords:** Age and sex disparities, Epidemiology, Preventive measures, Shoulder fractures, Trends

## Abstract

**Purpose:**

We aimed to identify temporal trends, seasonal changes and regional differences in shoulder fractures in Sweden during 2008–2022.

**Methods:**

Data from the Swedish National Board of Health and Welfare were used to assess incidence rates per 100,000 people, categorized by sex, age, and month.

**Results:**

Results showed an average of 17,496 fractures annually, with a decline in 2020 followed by a resurgence in 2021–2022. Elderly women, especially those over 65, had higher rates. Winter months exhibited increased incidence.

**Conclusions:**

Projection analysis indicated a gradual decrease in fractures over the next 15 years. Understanding these patterns can inform preventive strategies and resource allocation for shoulder fractures in Sweden.

## Introduction

Fractures of the shoulder, including clavicle, acromion, scapula and glenoid fractures, are prevalent orthopaedic injuries. Such injuries to upper extremities can significantly impact individuals’ well-being and independence [1].

Fractures in the shoulder commonly stem from traumatic incidents and are influenced by a range of factors including reduced bone density, muscle weakness, balance impairments, and age-related physiological shifts. Beyond inflicting physical discomfort and restricting mobility, these fractures heighten the likelihood of hospital admission and are associated with increased mortality. [2–4]. While treatments, such as surgery, exist for these fractures, concerted efforts should be directed towards reducing their incidence. Shoulder fractures are associated with social impact for patients and high healthcare costs [5–7]. As the elderly populations increase, monitoring of trends in shoulder fracture incidence using readily available data can enable stakeholders to identify groups at risk and tailor specific responses.

The aim of this study is to identify trends in incidence of shoulder fractures in Sweden, with a specific emphasis on sex, age, and temporal disparities using publicly available data.

## Methods

### Study design and data source

The study was designed as a population-based retrospective observational study. The main source of data was publicly available open-source data Swedish National Board of Health and Welfare (SNBHW) [8]. The SNBHW national patient register (NPR) contain information on all diagnoses for all individuals receiving treatment in Swedish hospitals. The NPR can be utilized for analyses of fracture incidence on a population basis. ICD-10 codes are reported per unique personal identification number and are registered once per year and diagnosis group in order to minimizing the risk of duplicate reporting. Population census data was obtained from Statistics Sweden [9].

### Study population

The study population was defined as individuals who were diagnosed with a shoulder fracture during the time periods of 2008–2021 and 2008–2019 while residing in Sweden. Data from two periods were included, January 1st, 2008, to December 31st, 2021, and January 1st, 2008, to December 31st, 2019, in order to adjust for the influence of the COVID-19 pandemic on diagnostic trends. Patients were defined in to two age groups, above and below the age of 65. The age of 65 was chosen as a cutoff point due to the higher incidence of fractures in this population owing to lower bone density, higher fall incidence and increased frailty. We did not include fractures amongst people below the age of 18.

The International Classification of Diseases, Tenth Revision (ICD-10) codes were used to identify shoulder fractures. All fractures of the shoulder and upper arm (ICD-10 codes S42) were included, and the cases were extracted from the NPR diagnoses register. The total number of cases were 262,444.

### Data analysis

Incidence of shoulder fractures per 100,000 person-years was calculated and then stratified by sex and predefined age groups. We used weights derived from the population distribution of standard population estimates to calculate age-specific population rates.We adjusted the population at risk on a yearly basis to account for the increased mortality during the COVID-19 pandemic, [9].

Poisson regression was used as the trend predictor for shoulder fracture incidence. Seasonal variances in shoulder fractures incidence were calculated by dividing annual number of shoulder fractures with the number of shoulder fractures during the four yearly seasons. Linear regression analysis was performed to determine sex-specific incidence rates. Data calculations and prediction analysis was done using SPSS (Version 25.2). We defined a P-value of < 0.05 as statistically significant.

### Ethical considerations

This study was exempt from ethical review due to the exclusive use of open source data.

## Results

### Fracture incidence

The yearly average during 2008 to 2022 was 17 496 (range 16 027–19 170) shoulder fractures in Sweden. There was no significant change in fracture incidence compared to previous years during 2008–2019. 2020 showed a significant drop in incidence with a subsequent increase during 2021–2022 (Table 1).

**Table 1:** The total number of fracture cases, incidence rates per 100,000 individuals, percentage changes in incidence rates from the previous year, and the statistical significance of these changes

Year	Men (*n*)	Women (*n*)	Both Sexes (*n*)	Men Incidence Rate/100,000	Women Incidence Rate/100,000	Both Sexes Incidence Rate/100,000	% Change in Both Sexes Incidence from Previous Year	Statistical Significance (*p*-value)	
2008	6 197	9 830	16 027	171	265	219	-	-	
2009	6 395	9 826	16 221	175	262	219	+ 0.0%	-	
2010	6 490	10 095	16 585	175	266	221	+ 0.9%	-	
2011	6 678	10 432	17 110	179	273	226	+ 2.3%	-	
2012	6 721	10 258	16 979	178	266	223	-1.3%	-	
2013	6 918	10 693	17 611	182	276	229	+ 2.7%	-	
2014	6 698	10 246	16 944	174	262	218	-4.8%	-	
2015	6 915	10 867	17 782	178	276	227	+ 4.1%	-	
2016	7 131	10 825	17 956	181	272	227	+ 0.0%	-	
2017	6 959	11 218	18 177	175	280	227	+ 0.0%	-	
2018	7 189	10 951	18 140	178	271	225	-0.9%	-	
2019	7 357	11 217	18 574	181	275	228	+ 1.3%	-	
2020	6 698	10 373	17 071	164	253	208	-8.8%	*p* < 0.001	
2021	6 958	11 139	18 097	169	270	219	+ 5.3%	*p* = 0.006	
2022	7 376	11 794	19 170	177	284	230	+ 5.0%	*p* = 0.012	

**Fig. 1:**
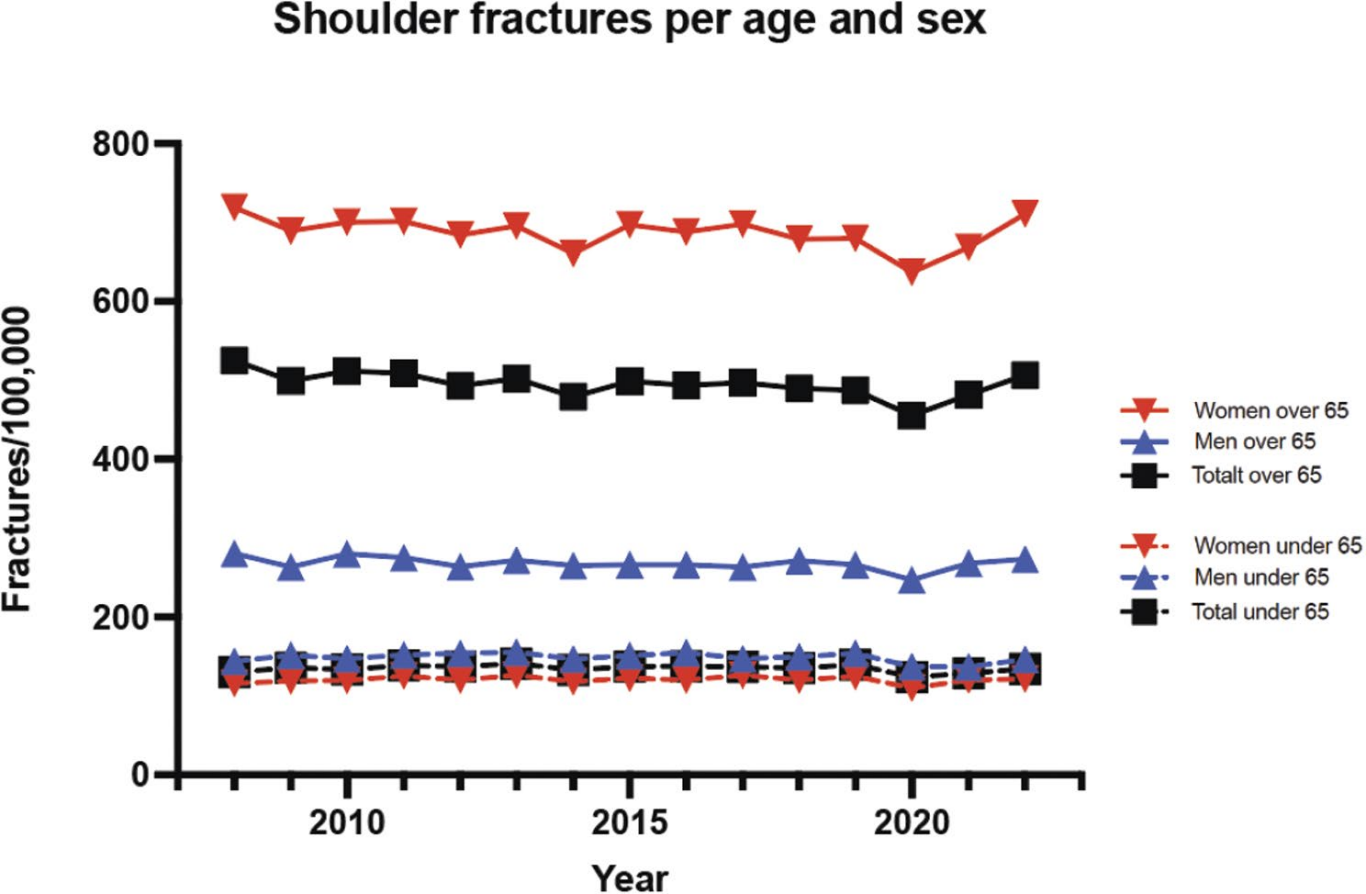
Distribution of age and sex in shoulder fracture incidence rates during the study period

Men (*p* < 0.001) and women over 65 (*p* < 0.001) years of age showed a significant decrease in shoulder fracture incidence during 2020 (Fig. 1).

### Age and sex distribution

**Fig. 2:**
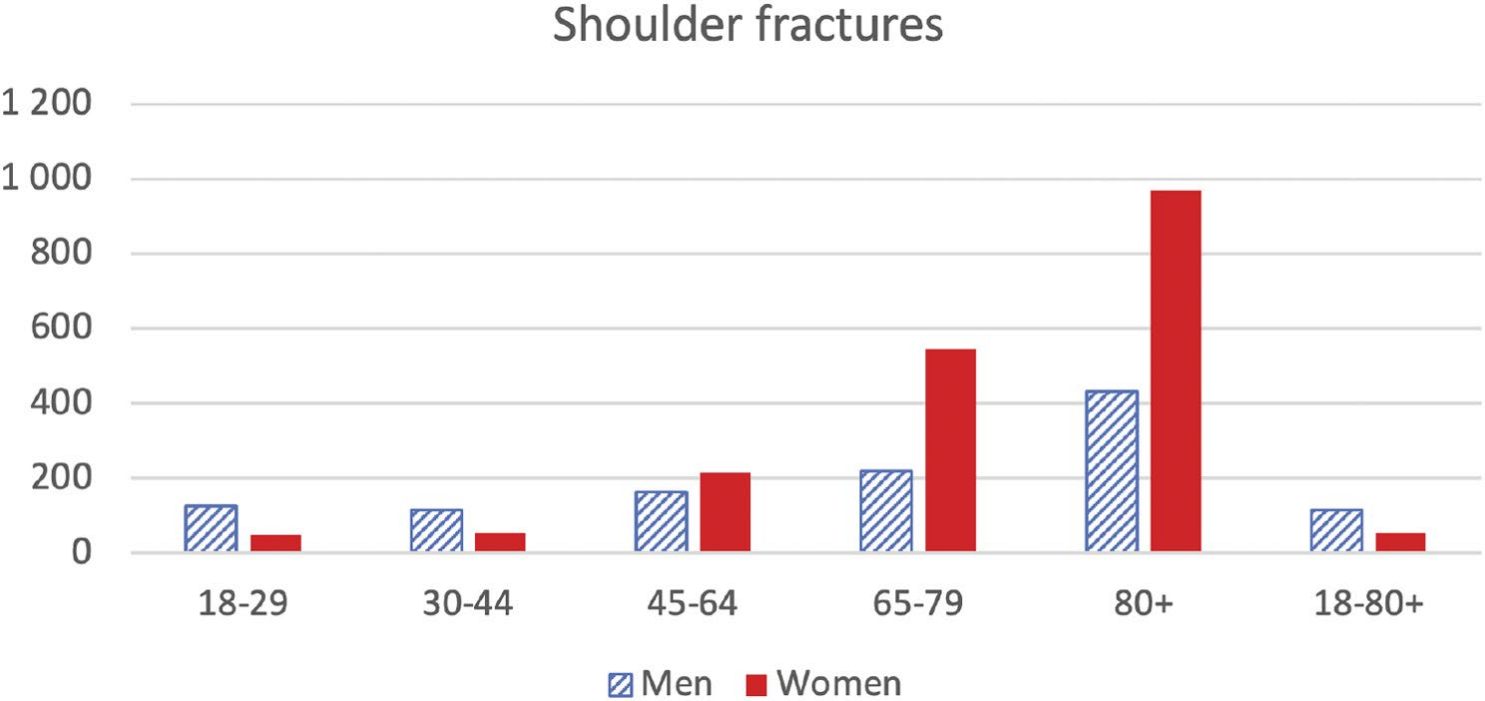
Shoulder fractures per age and sex

In women, the incidence of fractures increased steadily from the fifth decade to reach a peak of 967/100,000 per inhabitants in the 80 + age group. For men, the incidence topped at 431/100,000 per inhabitants in the 80 + age group (Fig. 2).

### Seasonal variance in fracture incidence

**Fig. 3:**
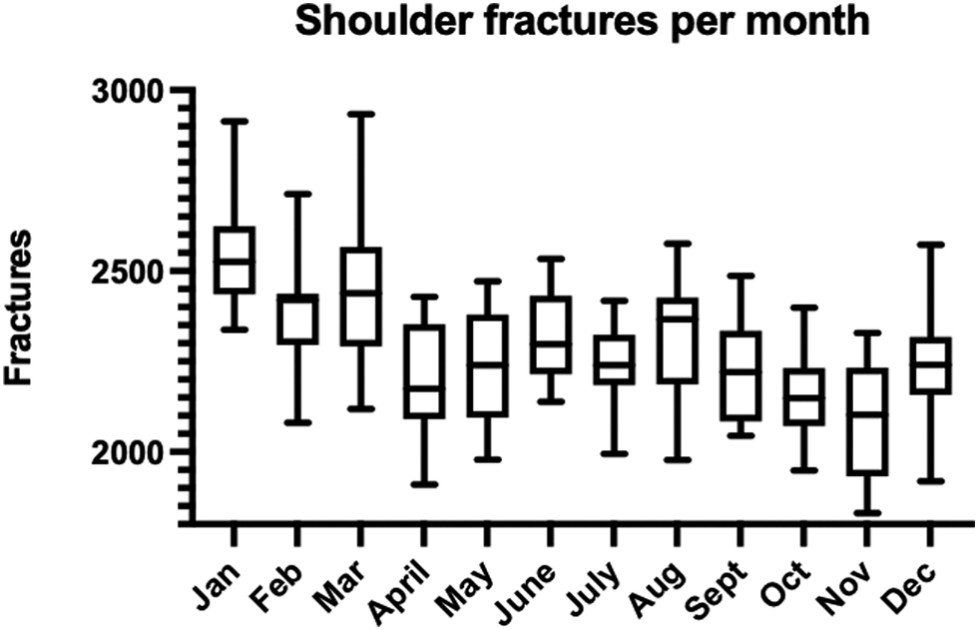
Box plots of the yearly distribution of shoulder fractures during 2008–2022

The late winter months and early spring (January-March) displayed higher rates of fractures compared to other months (Fig. 3).

**Fig. 4:**
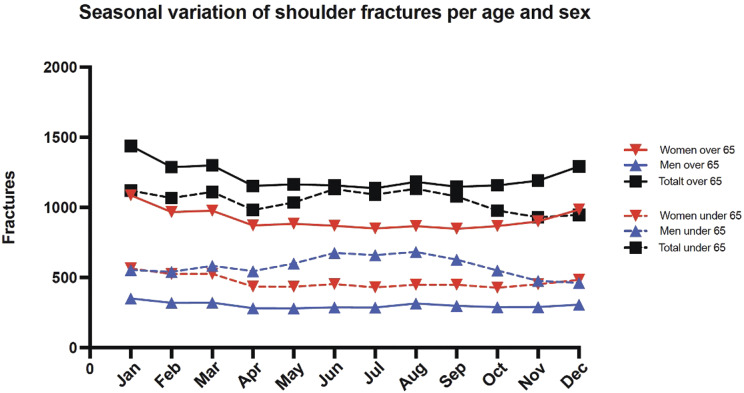
Seasonal distribution of shoulder fractures. Defined per age and sex. Mean is presented as average during 2008–2022

27% of fractures occurred in winter compared with 25%, 25% and 23% in spring, summer and autumn. Women over 65 years of age had a higher incidence rate during the winter months compared to other groups while men under the age of 65 had more fractures in the summer months (Fig. 4).

### Fracture projection analysis

**Fig. 5:**
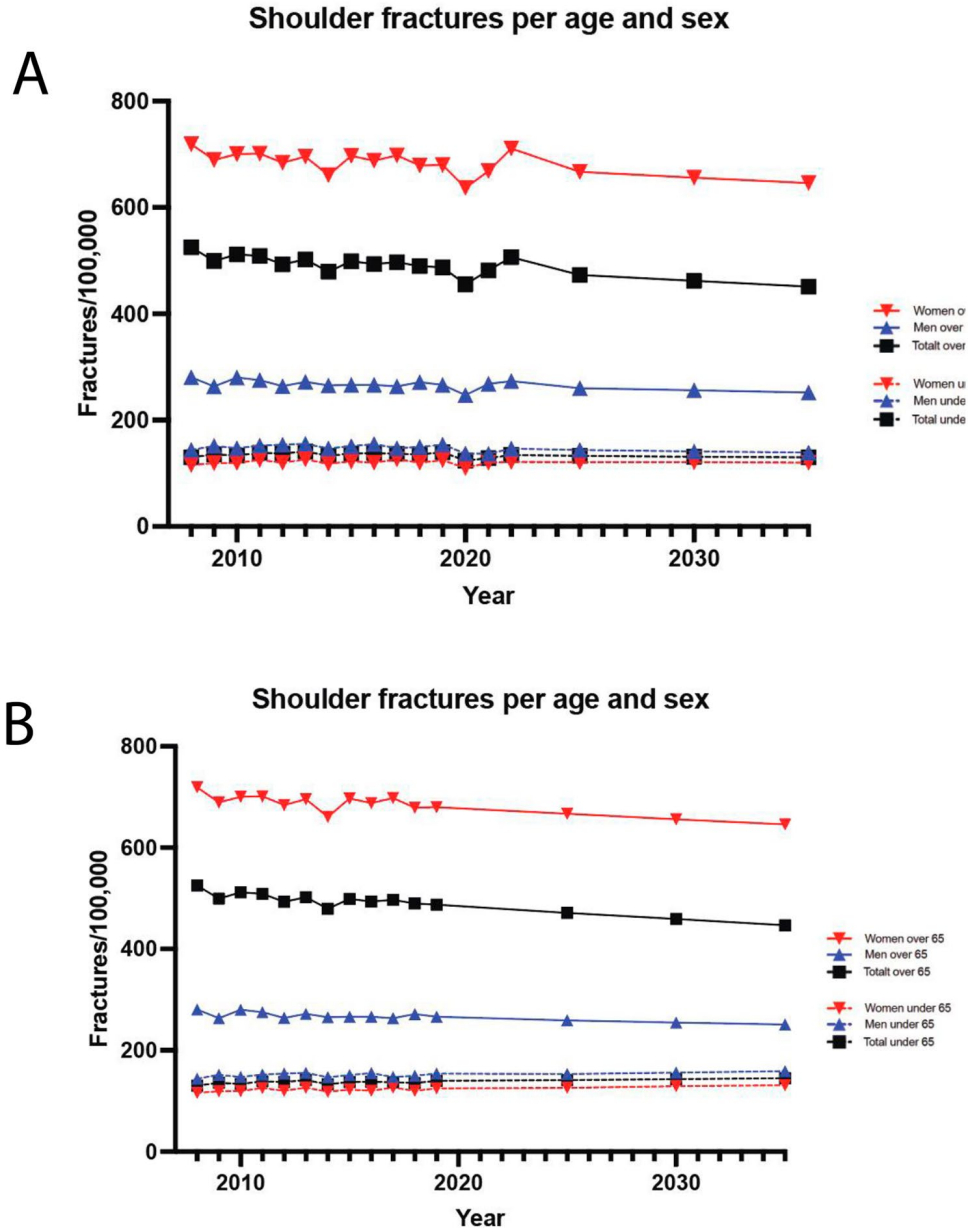
Future projection analysis of shoulder fracture incidence (**A**). Analysis excludes the pandemic years of 2020–2022 (**B**)

There is a decrease trend in shoulder fracture incidence during the next 15 years (Fig. 5A). Excluding the pandemic years of 2020–2021 in the projection analysis produced a less aggressive decline amongst the younger groups for 2025–2035 (Fig. 5B).

We compared projected changes in shoulder fracture incidence for 2025, 2030, and 2035 relative to 2022, with and without the inclusion of COVID-19 years (2020–2022). For individuals over 65, projections are consistent regardless of whether COVID-19 data is included, showing no statistically significant differences. In contrast, projections for those under 65 show a significant difference when COVID-19 years are excluded, with a higher projected incidence (Table 2).

**Table 2:** Future projection analysis of expected change in shoulder fracture incidence during 2025–2035 compared to 2022 with or without COVID years (2020–2022). Statistical significant differences between analyses are indicated

Year	Total over 65 COVID Years Included	Total over 65 COVID Years Excluded	Statistical Significance (*p*-value)	Total under 65 COVID Years Included	Total under 65 COVID Years Excluded	Statistical Significance (*p*-value)	
2025	93%	93	-	98%	105%	0.021	
2030	91%	91	-	98%	106%	0.017	
2035	89%	88	-	97%	108%	0.004	

## Discussion

The findings of this study show differences in distribution across age and sex groups, seasonal variations, and projected trends of shoulder fractures in Sweden. Men older than 80 years and women 65 years and older were two group at risk for shoulder fractures which is in line with previous data [10, 11]. These results were not surprising given that shoulder fractures are a common injury in elderly women due to osteoporosis, increased tendency for falls and higher frailty in this population [12, 13]. The next 15 years, according to our analysis, will display slightly lower incidence in shoulder fracture incidence in people over 65 years of age and in older women. The COVID-19 pandemic had also impacted the shoulder fracture incidence numbers in Sweden during 2020–2022, which affected the analysis.

There is another validated register for fractures in Sweden, the Swedish register for fractures, the ‘Svenska Frakturregistret’ [14]. However, the Swedish register for fractures suffers from poor data completeness as it is not mandatory for regions to participate in its reporting. The NPR includes all registered citizens in Sweden and therefore provides a more complete image of fracture incidence than the Swedish register for fractures and has been externally validated [15]. However, the NPR does not contain information about treatments or subtypes of fractures which hinders further analysis. Additional studies are therefore required to examine potential variations in patient characteristics or injury mechanisms among different subtypes of shoulder injuries. Unfortunately, due to the limitations in the available dataset, described above, this analysis was not feasible using our dataset.

The COVID-19 pandemic period (2020–2022) presented unique challenges that likely influenced the trends observed in shoulder fracture incidence, particularly among the elderly population. During this period, Sweden, like many other countries, experienced significant increases in mortality rates, particularly among those aged 65 and older, due to the direct and indirect effects of the pandemic. To account for the potential impact of this increased mortality on our analysis, we adjusted the population at risk on a yearly basis using age-specific mortality data.

It is important to recognize that the healthcare-seeking behavior and overall lifestyle changes during the pandemic, such as reduced physical activity and altered social interactions due to lockdowns and restrictions, may have contributed to the observed trends. Reductions in both incidence rates and number of shoulder fractures during 2020 has previously been reported in numerous publications in other studies [16–18]. Similar trends have been observed for other injuries and diseases during the pandemic [19]. However, to our knowledge this is the first nationwide observational study on shoulder fractures during the pandemic. Although Sweden notably avoided implementing stringent lockdown measures. This, coupled with alterations in activity levels, could account for the observed decline in fracture incidence during the pandemic years.

In our projection analysis, we observed a decrease in fracture incidence among the elderly population, which might seem counterintuitive given the expected increase in this demographic. This trend could be explained by several factors. Improved preventive measures, such as better osteoporosis management and fall prevention programs, alongside advancements in healthcare, likely contribute to reducing fracture rates [20, 21]. Additionally, behavioural changes, such as increased physical activity and better overall health among the elderly, may play a role in mitigating fracture risk. In our analysis, the exclusion of COVID-19 years significantly impacted the projected incidence of shoulder fractures in individuals under 65, with a notably higher expected incidence when these years were excluded. This suggests that the pandemic had a substantial effect on fracture trends in younger populations, likely due to changes in behaviour or healthcare access during this period. In contrast, projections for those over 65 remained consistent regardless of COVID-19 data inclusion, indicating that the pandemic had a less pronounced impact on this age group’s long-term fracture trends. However, caution is warranted in interpreting these projections as our projection model may not fully account for the impact of demographic changes. In particular the expected increase in elderly population which may skew incidence numbers [22].

The seasonal variance in fracture incidence, with a statistically significant increase during winter months. The higher incidence of fractures during the winter months can be attributed to several factors. In Sweden, winter is characterized by icy and slippery conditions, which increase the likelihood of falls, especially among the elderly. Additionally, reduced daylight hours and colder temperatures may limit outdoor activity, potentially leading to decreased physical fitness and balance, further heightening fracture risk. This seasonal trend is consistent with previous studies that have documented similar patterns [23, 24]. Further investigation into the reasons behind this seasonality is warranted in order to facilitate targeted preventive measures during the winter months to protect vulnerable populations, particularly the elderly.

While this study provides insights, certain limitations should be acknowledged. The retrospective observational nature of the analysis relies on accurate and complete reporting in publicly available healthcare records. We were also not able to distinguish between certain subgroups of shoulder fractures as the ICD-10 codes we analyzed does not distinguish between fractures. Nevertheless, we believe our findings provide important data in the expected incidence of shoulder fractures in the population in Sweden using easily available and stratified data.

## Conclusion

In conclusion, this study found differences in sex, age and seasonal variance related to shoulder fractures in Sweden. Elderly women are a particular vulnerable group which are at risk for shoulder fractures. Future analysis shows slightly decreasing incidence of shoulder fractures. There was no significant effect of the COVID-19 pandemic on future shoulder fracture incidence.

## Data Availability

Data is available from the corresponding author on reasonable request. Open source base data can be obtain from (https://www.socialstyrelsen.se/en/).
